# Enhancement of Magnetorheological Fluids with Size and Morphology—Optimized Fe_3_O_4_ Nanoparticles: Impacts on Rheological Properties and Stability

**DOI:** 10.3390/ma17122838

**Published:** 2024-06-11

**Authors:** Liwei Xu, Guangdong Zhou

**Affiliations:** College of Chemistry, Jilin University, No.2699 Qianjin Street, Changchun 130012, China; xulw21@mails.jlu.edu.cn

**Keywords:** carbonyl iron, magnetorheological fluids, additive

## Abstract

In this study, we synthesized Fe_3_O_4_ nanoparticles (Fe_3_O_4_ NPs) of varying sizes and morphologies using the solvothermal method and incorporated them as additives into carbonyl iron magnetorheological fluids (CI-MRFs). We tested the shear stress, yield stress, viscosity and storage modulus of the MRFs using a magnetorheometer to investigate how the size and morphology of Fe_3_O_4_ NPs influence the performance of MRFs. Our results indicate that the size of the additive nanoparticles significantly enhances the MR properties of MRFs more than their morphological attributes. This enhancement results from optimizing and stabilizing the CI magnetic chain structure of the nanoparticles in the presence of a magnetic field. Specifically, MRFs with Fe_3_O_4_ NPs averaging 250 nm in size exhibit higher yield stress and storage modulus and show increased resistance to shear strains. Although the nanoparticle morphology has a modest effect on the rheological properties of MRFs, hexahedral and octahedral particles can enhance rheological properties through increased internal friction compared to spherical particles. Additionally, Fe_3_O_4_ NPs of different sizes and morphologies improve the sedimentation stability of MRFs, with those around 250 nm being particularly effective at slowing down sedimentation. Both hexahedral and octahedral Fe_3_O_4_ NPs slow down sedimentation more effectively than spherical Fe_3_O_4_ NPs. This paper investigates the rheological properties of CI-MRFs by controlling the additive particle size and morphological features, providing a research foundation for the design and optimization of MRFs.

## 1. Introduction

Magnetorheological fluids (MRFs) are a distinct class of smart materials composed of a non-magnetic carrier fluid, magnetic particles and specific additives [1,2,3]. A unique characteristic of MRFs is their ability to form chain-like microstructures within the magnetic particles, modulated by an external magnetic field [4]. This results in controllable rheological properties, allowing precise modulation of the yield stress, viscosity and sedimentation rate of the MRFs. Consequently, MRFs are employed in diverse applications, including vibration dampers [5], sensors [6], seals [7], polishing [8] and biomedical devices [9,10].

In recent decades, extensive research has prepared various magnetic particles, such as carbonyl iron (CI), Fe_3_O_4_ and CrO_2_ [11]. Among these, CI has been predominantly used in MRFs due to its high permeability, soft magnetic properties and cost-effectiveness. However, CI’s high density predisposes it to sedimentation, owing to the significant density disparity between CI and the carrier fluid.

Researchers have employed various strategies to address the deposition issue in CI, such as incorporating surfactants [12], polymer nanoparticles [13] and organic or inorganic coatings on CI [14,15]. These approaches aim to decrease the density of particles and optimize their surface structure to mitigate the aggregation and deposition problems associated with CI-based MRFs. However, these methods often result in a decline in MRF performance while attempting to solve this problem. On the other hand, introducing magnetic nanoparticles has proven to be an effective solution for alleviating the aggregation and deposition issues in magnetic fluids, thereby providing a robust technical approach to enhance the overall performance of MRFs.

In recent years, Fe_3_O_4_ magnetic particles have gained considerable attention as potential additives in CI-MRFs, primarily due to their ability to adjust their particle size, morphology and density [16,17]. However, there is a scarcity of research investigating the specific influence of additive particle size and morphology on MRF performance.

Based on previous work on Fe_3_O_4_-halloysite composites and their concentrations [18], we further investigated the impact of Fe_3_O_4_ particle size and morphology on CI-MRF. This study synthesized Fe_3_O_4_ NPs using a solvothermal method, varying the particle sizes and morphologies by employing different surfactants. Subsequently, we prepared CI-Fe_3_O_4_-MRF by incorporating Fe_3_O_4_NPs as additives into CI-MRF and evaluated its properties. This study comprehensively analyzes how particle size and morphology influence CI-MRF, utilizing the existing theoretical model. This approach establishes a robust research foundation and offers technical support for the design and optimization of CI-MRF.

## 2. Materials and Methods

All reagents used in the experiments were analytically pure. The synthesis of Fe_3_O_4_ NPs was carried out following literature protocols [19,20,21,22], adjusting the type and amount of surfactants accordingly.

Two MRFs were prepared: the first, a monodisperse phase MRF, consisted of 50 wt% carbonyl iron particles (CIPs) and dimethicone oil (Aladdin, viscosity: 100 mPa*s); the second, a bi-disperse phase MRF, included Fe_3_O_4_ (1 wt%) NPs, CIPs (49 wt%) and dimethicone oil. Continuous stirring and ultrasonic dispersion were employed to ensure the homogeneous and adequate dispersion of both MRFs.

Morphological observation and energy spectrum analysis of CI and Fe_3_O_4_ were performed using a cold field emission electron microscope (SEM) (SU8020, Hitachi, Tokyo, Japan) and a high-resolution transmission electron microscope (TEM) (Tecnai G2, FEI, Hillsboro, OR, USA). Particle size statistics for both materials were obtained using the SEM images and analyzed using particle size statistics software (Nano Measurer Ver1.2). The crystal structure of the synthesized Fe_3_O_4_ was characterized using an X-ray diffractometer (XRD) (Empyrean, PANalytical B.V, Almelo, The Netherlands). Magnetic properties of CI and Fe_3_O_4_ were investigated with a Magnetic Property Measurement System (MPMS) (MPMS-XL7, Quantum Design, San Diego, CA, USA). The rheological properties of the MRFs were studied using a magnetorheometer (MCR-302, Anton Paar, Graz, Austria).

## 3. Results

**Figure 1:**
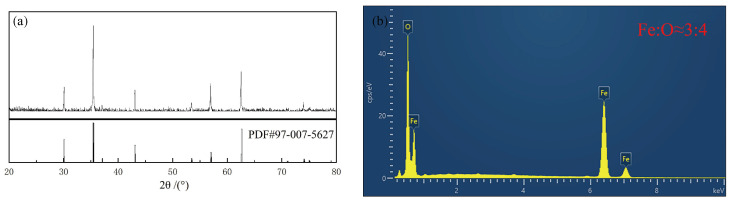
XRD (**a**) and EDS (**b**) patterns of Fe_3_O_4_.

The composition of the obtained sample was analyzed using X-ray diffraction (XRD), with the results presented in Figure 1a. When compared with the standard Fe_3_O_4_ diffraction pattern [23], an overlap in the positions of characteristic peaks was observed, confirming that the sample was pure Fe_3_O_4_. Further validation came from the energy-dispersive X-ray spectroscopy (EDS) analysis shown in Figure 1b, where the elemental ratio of Fe to O was approximately 3:4, corroborating the synthesis of Fe_3_O_4_.

**Figure 2:**
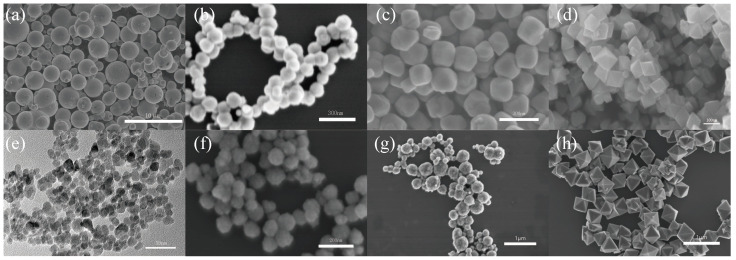
SEM images of the (**a**) CI, (**b**) 120 nm spherical Fe_3_O_4_, (**c**) 120 nm octahedral Fe_3_O_4_, (**d**) 120 nm hexahedral Fe_3_O_4_, (**e**) TEM-10 nm Fe_3_O_4_, (**f**) 80 nm Fe_3_O_4_, (**g**) 250 nm Fe_3_O_4_, (**h**) 500 nm Fe_3_O_4_.

SEM and TEM images of CI and Fe_3_O_4_ are presented in Figure 2. Specifically, Figure 2a displays carbonyl iron particles that are spherical with an average diameter of approximately 3 µm. Figure 2b–d depict spherical, hexahedral and octahedral Fe_3_O_4_ particles, each with an average size of about 120 nm. Figure 2e illustrates irregularly shaped particles averaging 10 nm in size. Figure 2f,g show spherical particles with average diameters of approximately 80 nm and 250 nm, respectively. Lastly, Figure 2h reveals octahedral particles with an average size of about 500 nm.

**Figure 3:**
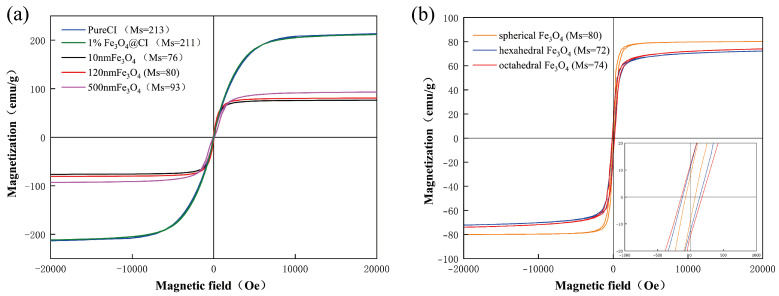
Magnetic hysteresis loops of (**a**) the CI and Fe_3_O_4_ NPs with different particle sizes, (**b**) 120 nm Fe_3_O_4_ NPs with different morphology.

Figure 3 illustrates the magnetic hysteresis loops of the samples, indicating that both CI and Fe_3_O_4_ NPs exhibit characteristics of soft magnetic materials. Notably, the saturation magnetization of Fe_3_O_4_ NPs increases with particle size. Furthermore, Figure 3a reveals that the saturation magnetization values for CI and CI with 1 wt% Fe_3_O_4_ (Fe_3_O_4_@CI) are 213 emu/g and 211 emu/g, respectively. This similarity in values suggests that incorporating Fe_3_O_4_ does not significantly alter the saturation magnetization of CI. Figure 3b illustrates the magnetic hysteresis loops of Fe_3_O_4_ NPs with three distinct morphologies but equivalent particle sizes. The data reveal that spherical Fe_3_O_4_ demonstrates the highest magnetism, measuring 80 emu/g, whereas hexahedral Fe_3_O_4_ exhibits the weakest magnetism at 72 emu/g.

**Figure 4:**
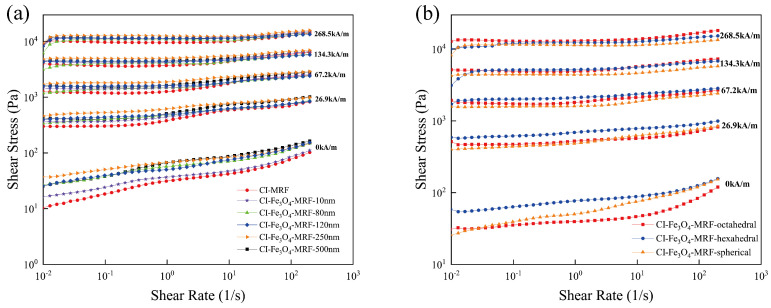
(**a**) Flow curves of CI-MRF and CI-Fe_3_O_4_-MRFs with different particle sizes, (**b**) Flow curves of CI-Fe_3_O_4_-MRFs with different morphology.

Rheological testing of CI-MRFs and CI-Fe_3_O_4_-MRFs was conducted using a magnetorheometer in a controlled shear rate mode, with magnetic field strengths ranging from 0 to 268.5 kA/m and shear rates from 0.01 to 200 s^−1^. The results are depicted in Figure 4. The morphologies of the Fe_3_O_4_ NPs used in the study are consistently spherical across different particle sizes.

Figure 4a presents the shear stress versus shear rate curves for MRFs containing CI and Fe_3_O_4_ NPs with varying particle sizes. Without an external magnetic field, CI-MRFs and CI-Fe_3_O_4_-MRFs behave as Newtonian fluids. Upon application of the magnetic field, they transition to Bingham fluids, characterized by distinct yield stresses. As illustrated in Figure 4a, incorporating Fe_3_O_4_ NPs into MRFs enhances their shear stress, suggesting that Fe_3_O_4_ NPs fill the voids within the CI magnetic chains, reinforcing them. Additionally, as the magnetic field strength increases, the range of shear stress that remains stable at a given shear rate broadens. This phenomenon is attributed to the interaction among adjacent magnetic chains, which coalesce to form larger, more robust magnetic structures. Specifically, the CI-Fe_3_O_4_-MRF with 250 nm particles exhibits higher shear stress under identical conditions, indicating that this optimal particle size effectively bridges gaps between CI magnetic chains and strengthens the magnetic chain structure.

Compared to spherical Fe_3_O_4_ NPs, hexahedral Fe_3_O_4_ NPs stabilize the magnetic chains more effectively, with octahedral particles being the next most effective. This enhanced stability is presumably due to the polyhedral morphology of these particles, which provides greater surface area for friction and consequently improves stress transfer between magnetic chains [24]. This effect is akin to roughening the surface of CI particles, thereby increasing friction and reinforcing the overall structure [25].

**Figure 5:**
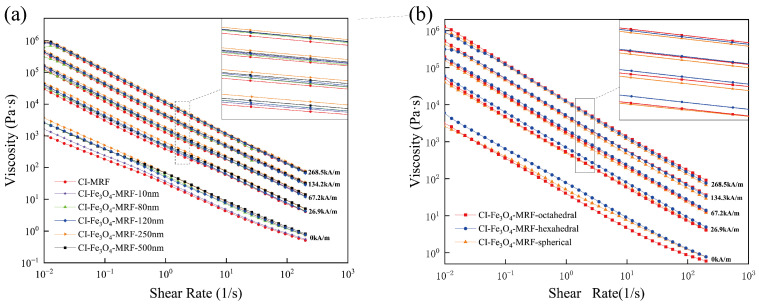
(**a**) Viscosity curves of CI-MRFs and CI-Fe_3_O_4_-MRFs with different particle sizes, (**b**) Viscosity curves of CI-Fe_3_O_4_-MRFs with different morphology.

Figure 5 illustrates the shear viscosity versus shear rate curves for both CI-MRFs and CI-Fe_3_O_4_-MRFs. As depicted, the viscosity of these MRFs, similar to shear stress, increases with the magnetic field intensification. Concurrently, an increase in shear rate leads to decreased shear viscosity, demonstrating a pronounced shear-thinning behavior. Although adding Fe_3_O_4_ NPs results in a slight increase in MRF viscosity, the change is relatively modest. In engineering applications, the zero-field viscosity at a 100 s^−1^ shear rate is commonly used as an evaluation criterion [26]. The zero-field viscosities for CI-MRFs to CI-Fe_3_O_4_-MRF-500 nm formulated in this study were measured at 0.77, 0.85, 1.16, 1.22, 1.23 and 1.35 Pa·s, respectively. These results indicate that zero-field viscosity increases with an increase in particle size. For different morphologies, the zero-field viscosities of spherical, hexahedral and octahedral particles were found to be 1.22, 1.27 and 0.85 Pa·s, respectively. Notably, incorporating of octahedral Fe_3_O_4_ NPs seems to reduce the zero-field viscosity compared to the other morphologies. The complexation of octahedral Fe_3_O_4_ NPs with CI under zero-field conditions decreases interparticle friction [27]. This decrease can be attributed to a reduction in the contact area between particles, ultimately leading to a decrease in viscosity. Generally, a broader range of viscosity values enables the MRFs to cater to a wider array of application requirements.

To obtain the yield stress of the MRFs, the flow curves were fitted using the Herschel-Bulkley (1), Bingham (2) and Casson (3) models with the following equations:

(1) $$\tau =\tau_{y}+K{\dot{\gamma }}^{n}$$

(2) $$\tau =\tau_{y}+\eta_{0}\dot{\gamma }$$

(3) $$\tau^{1/2}=\tau_{y}^{1/2}+\eta_{\infty }^{1/2}{\dot{\gamma }}^{1/2}$$

where τ is the shear stress, $\tau_{y}$ is the yield stress, K is the consistency index, n is the flow index, $\eta_{0}$ is the zero shear viscosity and $\eta_{\infty }$ is the Casson viscosity.

**Figure 6:**
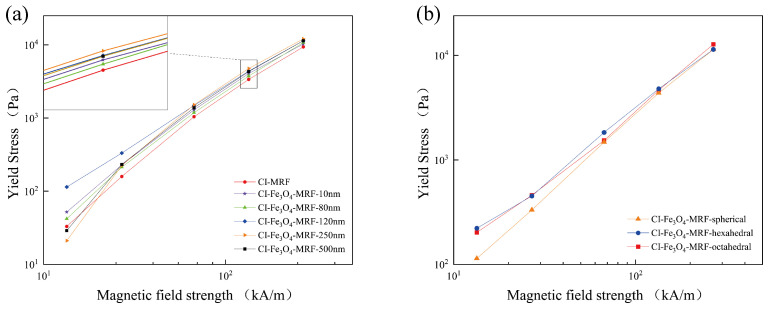
(**a**) Yield stresses of CI-MRFs and CI-Fe_3_O_4_-MRFs with different particle sizes, (**b**) Yield stresses CI-Fe_3_O_4_-MRFs with different morphologies.

The parameters for the three evaluated models are summarized in Table 1. Based on the R^2^ values, the Herschel-Bulkley model demonstrates the best fit, offering a more accurate depiction of the rheological properties than the Casson and Bingham models. Consequently, the Herschel-Bulkley model was employed to calculate the yield stress for each MRF, with the results presented in Figure 6. It is evident from Figure 6 that the dynamic yield strength of the MRFs increases progressively with the escalation of magnetic field strength.

In general, the relationship between the yield stress and the magnetic field strength can be expressed by the Equation (4).

(4) $$\tau_{y}\propto H^{\alpha }$$

Some studies [28] have reported that the relationship between yield stress and magnetic field strength at low magnetic field strength can be expressed by Equation (5):

(5) $$\tau_{y}\propto H^{2}$$

At intermediate magnetic field strengths, expressed in Equation (6):

(6) $$\tau_{y}\propto H^{3/2}$$

At higher magnetic field strengths, the yield stress is independent of the magnetic field strength and depends on the saturation magnetic field strength of the particles Ms, as shown in Equation (7).

(7) $$\tau_{y}\propto M_{s}^{2}$$

It has been shown that the value of α in Equation (4) does not perfectly fit the above theory when it is affected by different temperatures or interparticle interactions [29]. We found that the α value of CI-MRF can perfectly fit the above theory. However, after the addition of Fe_3_O_4_NPs, the yield stress data of some particle sizes and morphologies show differences. At lower magnetic field strengths, the α values of CI-Fe_3_O_4_-MRF-120 nm and 500 nm deviate by a significant amount from the theoretical values, which are 1.6 and 2.3, respectively; at intermediate magnetic field strengths, the relationship between the yield stresses and the magnetic field strengths can better follow the theoretical expectations, both being about 1.5. This is because adding the Fe_3_O_4_NPs generates additional interactions, leading to deviations from the correlation with the theoretical equations [29]. Concerning particle size, we found that the above theoretical equations do not fit the MRF flow curves well with the addition of octahedral and hexahedral Fe_3_O_4_. This is due to the simplified extrapolation using spherical shapes during the derivation of the theoretical equations, which deviates from the experiments in actual experiments due to the particle morphology, which increases the drag force during particle movement. Nevertheless, in this study, CI-Fe_3_O_4_-MRF-250 nm and CI-Fe_3_O_4_-MRF-hexahedral show high yield stress under different magnetic fields.

**Figure 7:**
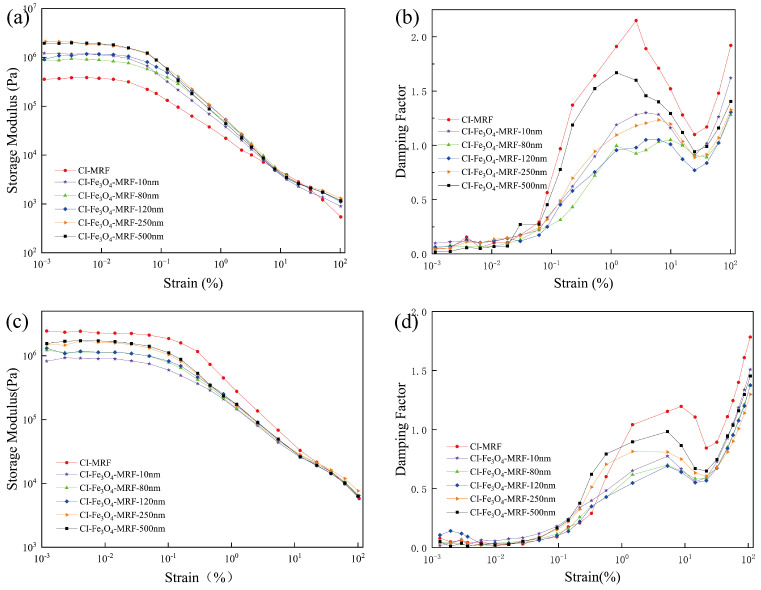
The storage modulus (G′), damping factor versus shear strain of CI-MRFs and CI-Fe_3_O_4_-MRFs with different particle sizes in 67.2 kA/m (**a**,**b**) and 268.5 kA/m (**c**,**d**) magnetic fields.

**Figure 8:**
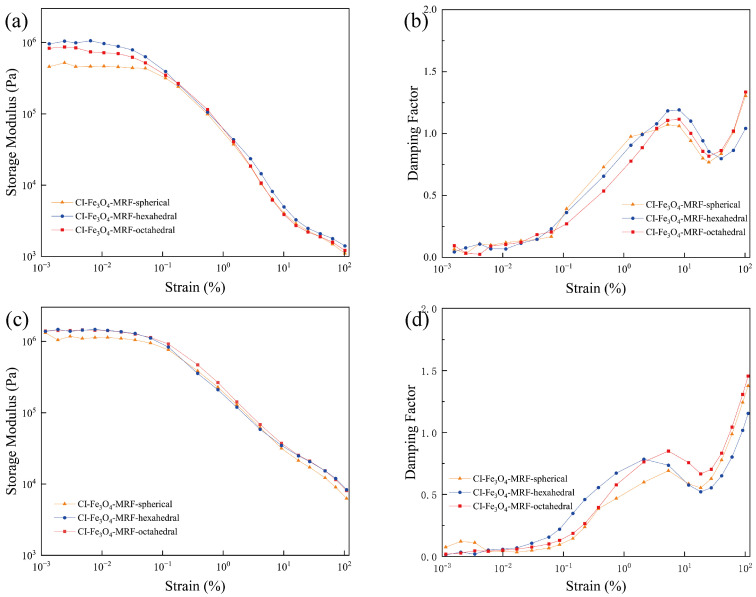
The storage modulus (G′), damping factor versus shear strain of CI-Fe_3_O_4_-MRFs with different morphologies in 67.2 kA/m (**a**,**b**) and 268.5 kA/m (**c**,**d**) magnetic fields.

Meanwhile, we tested the MRFs by amplitude scanning in order to detect their dynamic behavior. The results are shown in Figure 7 and Figure 8. The storage modulus (G′) represents the elastic property of the MRFs. The damping factor is the ratio of the loss modulus (G″) to the storage modulus (G′), which indicates whether the material exhibits elasticity or viscosity. It behaves as elasticity when the damping factor < 1, and when the damping factor > 1, it is viscous.

Figure 7 illustrates that the linear viscoelastic range (LVE) of MRFs extends with increasing magnetic field strength. At lower magnetic field strengths, the storage modulus (G′) of CI-Fe_3_O_4_-MRFs shows an initial increase compared to CI-MRFs. Notably, the storage modulus (G′) remains constant over a certain strain range before decreasing as strain further increases. This behavior is particularly evident in CI-Fe_3_O_4_-MRF-250 nm, as shown in Figure 7b, where a higher storage modulus is maintained, indicating that its magnetic chain structure can endure larger strains.

Under high magnetic field strengths, CI-MRFs display the highest storage modulus, and a trend emerges where the magnitude of storage modulus (G′) diminishes with decreasing particle size. This observation is attributable to CI’s higher saturation magnetization intensity than Fe_3_O_4_, and that Fe_3_O_4_’s saturation magnetization intensity reduces with decreasing grain size. Consequently, incorporating Fe_3_O_4_ NPs reduces the overall saturation magnetization intensity of the material, aligning with the results shown in Figure 3a. Further analysis of Figure 7b,d suggests that adding Fe_3_O_4_ NPs of an appropriate particle size can enhance the material’s resistance to strain.

Figure 8 displays the amplitude scan results for CI-Fe_3_O_4_-MRFs with various morphologies. Notably, the CI-Fe_3_O_4_-MRF with hexagonal morphology exhibits a higher storage modulus (G′) across different magnetic fields than MRFs with other morphologies. While the magnetic chain structures of the three MRF types influence their strain resistance to some extent, it is less significant than the effect of grain size. However, spherical Fe_3_O_4_ NPs are less effective in enhancing performance than their hexahedral and octahedral counterparts. Combined with Figure 3, it is evident that the hexahedral Fe_3_O_4_ exhibits the weakest magnetism, while CI-Fe_3_O_4_-MRF-hexahedral demonstrates the highest storage modulus. This distinctly highlights the enhancing effect of the hexahedral morphology on the MRFs.

One of the most essential properties of MRFs is sedimentation stability, which is a natural sedimentation phenomenon caused by the density difference between the carrier fluid and the particles. The sedimentation ratio (SR) is calculated from Equation (8):

(8) $$SR=(1-\frac{H_{s}}{H_{t}})*100\%$$

where H_*s*_ is the height of the supernatant and H_*t*_ is the overall height of the MRFs.

**Figure 9:**
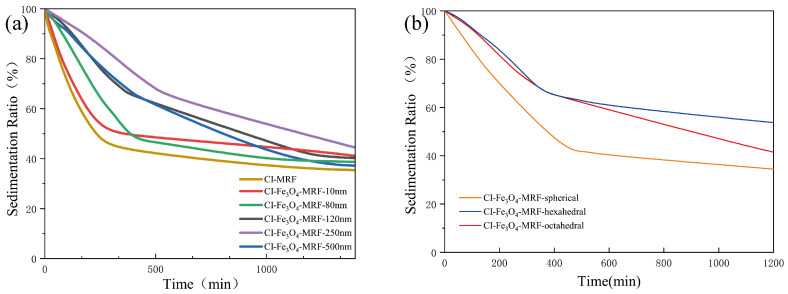
(**a**) Sedimentation ratios of CI-MRF and CI-Fe_3_O_4_-MRF with different particle sizes, (**b**) Sedimentation ratios of CI-Fe_3_O_4_-MRF with different morphologies.

Regulating the sedimentation process of MRFs is crucial, as sedimentation typically accompanies a reduction in yield stress. Figure 9a demonstrates that CI-Fe_3_O_4_-MRFs exhibit a pronounced delay in sedimentation compared to CI-MRFs. This is particularly notable in CI-Fe_3_O_4_-MRF-250 nm, where the sedimentation time is significantly extended, and the sedimentation rate is markedly reduced. Additionally, as shown in Figure 9b, hexahedral and octahedral Fe_3_O_4_ NPs are more effective in mitigating the sedimentation of MRFs compared to spherical Fe_3_O_4_ NPs.

## 4. Discussion

The rheological properties and sedimentation stability of bi-dispersed phase MRFs are notably improved by including Fe_3_O_4_ NPs with suitable particle size and morphology. The enhancement effect of nanoparticles on MRFs can be briefly analyzed using the following mechanism.

**Figure 10:**
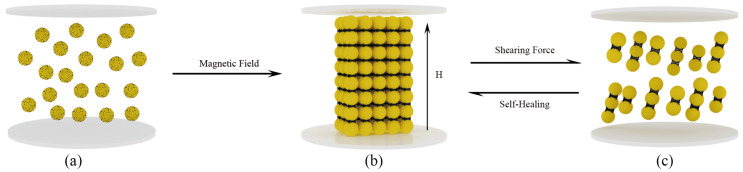
Shear modeling of CI-Fe_3_O_4_ bi-dispersed phase MRFs. (**a**) MRFs without magnetic field (**b**) MRF with magnetic field (**c**) MRFs after shear thinning.

CIPs in CI-MRFs exhibit random dispersion within the liquid phase when no magnetic field is applied. The incorporation of Fe_3_O_4_ NPs, which possess notable surface activity, small dimensions and low remanent magnetization, leads to the adsorption of these NPs onto the surfaces of the CIPs, as shown in Figure 10a. Upon applying a magnetic field, the CIPs and Fe_3_O_4_ NPs align to form chain-like structures, with the Fe_3_O_4_ NPs occupying the spaces between the CIPs [30]. As the strength of the magnetic field increases, these chains interlock, creating a more robust and stable columnar structure, as illustrated in Figure 10b.

The formation of this structure significantly impacts the characteristics of the MRFs, including yield stress, viscosity and storage modulus, all notably enhancing in response to a stronger magnetic field. Under shear conditions, magnetic chains undergo disruption and reconfiguration. At lower shear rates, these chains can reorganize, effectively maintaining their structure. Conversely, as depicted in Figure 10c, the chains disintegrate and disperse at elevated shear rates, complicating their reformation [31]. In this scenario, shear stress initially levels off before escalating sharply, viscosity consistently decreases and the storage modulus experiences a rapid decline after exceeding the linear viscoelastic region (LVE).

Furthermore, the impact of NPs on void filling varies with their sizes. Larger NPs are less effective in enhancing the structural integrity and permeability of magnetic chains due to their reduced filling density [32]. Conversely, smaller NPs can efficiently occupy voids, boosting structural strength at low shear strains. However, they act more like lubricants at higher shear strains due to their diminished magnetization strength and diminutive size. For instance, CI-Fe_3_O_4_-MRF with 10 nm particles shows low shear stress and viscosity alongside a high storage modulus within a limited strain range; however, its storage modulus swiftly declines as the strain increases. On the other hand, samples with moderate NPs sizes, like CI-Fe_3_O_4_-MRF-120 nm and 250 nm, preserve their elastic characteristics under higher strains, maintaining elevated yield stress and storage modulus.

In order to theoretically and accurately explain the chain structure model of bi-dispersed phase MRFs with different particle sizes, a simple single chain structure model may introduce significant errors. Therefore, we adopt Equation (9) [33] for a more in-depth theoretical analysis of the chain structure of bi-dispersed phase MRFs. Here we briefly replace the macroscopic shear yield stress with the shear yield stress.

(9) $$\begin{array}{c} \bar{\tau }=\tau_{0}+\int_{-\pi /2}^{\pi /2}\frac{2PA\mu_{0}\phi R_{1}^{3}R_{2}^{3}\chi^{2}H^{2}\cos^{4}\theta \sin\theta }{{\left(R_{1}+R_{2}+2t+\delta \right)}^{3}}\\ \times \left(5\cos^{2}\theta -1\right)\frac{1}{\sqrt{2\pi }\sigma }e^{-\frac{{\left(\theta -\mu \right)}^{2}}{2\sigma^{2}}}d\theta \end{array}$$

where $\bar{\tau }$ is macroscopic shear yield stress, $\tau_{0}$ is viscous damping force of the MRF under zero field, P is the number of particle chains in the unit cross-sectional area, A can be considered a constant, $\mu_{0}$ is the vacuum permeability, ϕ is the volume fraction of magnetic particles, R_1_, R_2_ are the diameters of the two magnetic particles, χ is magnetization rate, t is the thickness of the oil film, δ is gap between the adjacent particles in the chain, μ is the curve distribution center and σ represents the concentration degree of the relative distribution center of the particle chain.We can evaluate the effects of Fe_3_O_4_ NPs by analyzing the changes in parameters resulting from their addition. As depicted in Figure 10b, incorporating nanoparticles leads to an increase in the MRF’s overall volume fraction, consequently densifying the magnetic chain structure. This densification results in elevated values of ϕ and a reduction in δ. Simultaneously, the equation R_1_R_2_/(R_1_ + R_2_ + 2t + δ) reaches a maximal value, indicating the presence of an optimal particle size for the MRFs. The addition of 250 nm Fe_3_O_4_ NPs is close to the optimal particle size in the MRFs, exhibiting the best rheological performance.

Without a magnetic field, particles are randomly distributed on the CI surface, with octahedral particles potentially creating a sea urchin-like morphology. This structure increases the gaps between particles while reducing contact areas, particularly in comparison to spherical and hexahedral shapes. Consequently, octahedral particles exhibit lower viscosity without a magnetic field than their counterparts [34].

**Figure 11:**
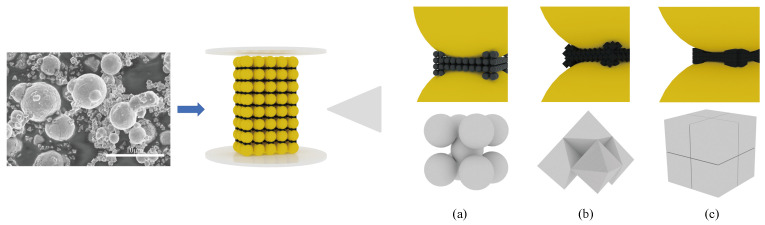
Void filling modeling of CI by Fe_3_O_4_ NPs with different morphologies. (**a**) spherical, bct lattice, (**b**) octahedral, (**c**) hexahedral.

When subjected to an external magnetic field, NPs tend to cluster at the connections of magnetic chains, with the strength of the chain influenced by the morphology of Fe_3_O_4_ NPs and their arrangement within the chains. Figure 11a–c illustrates the arrangement of Fe_3_O_4_ NPs of different shapes within magnetic chains [35]. The BCT lattice arrangement [36], shown in Figure 11a for spherical NPs is identified as the most effective in terms of space-filling. However, the denser arrangements of octahedral and hexahedral particles, depicted in Figure 11b,c, exhibit larger contact areas, reducing the efficacy of spherical friction compared to the other shapes. Meanwhile, the rolling friction generated by spherical particles is inferior to the sliding friction of particles with regular shapes [37]. This frictional dynamic is essential for enhancing the stability of magnetic chain structures and improving the efficiency of stress transmission. Therefore, regarding rheological performance, the yield stress and the storage modulus of CI-Fe_3_O_4_-MRFs with octahedral and hexahedral shapes are superior to those with spherical particles. The interaction between particles and magnetic chains further distinguishes the hexahedral assemblies as more conducive to formation than the octahedral embedded combinations, thereby endowing MRFs containing hexahedral particles with a higher storage modulus [38]. However, at strong magnetic fields, the high magnetization of carbonyl iron reduces the influence of NP morphology, resulting in similar rheological properties among Fe_3_O_4_ NPs of different morphologies.

Sedimentation in MRFs is primarily driven by gravity, which the carrier fluid alone cannot effectively resist. In order to enhance sedimentation stability, one strategy is to reduce the particle density, while another is to strengthen the interaction between particles and the carrier fluid. The addition of nanoparticles helps to lower the overall density of the mixture, thereby improving the sedimentation stability of MRFs. Larger nanoparticles, such as those in CI-Fe_3_O_4_-MRF-120 nm and 250 nm, enhance stability by occupying more space and providing more excellent resistance. The morphology of nanoparticles also plays a role; octahedral and hexahedral particles, unlike spherical ones, offer better resistance during sedimentation, contributing to stability by increasing the interactions between particles [39].

## 5. Conclusions

In this study, we prepared Fe_3_O_4_ NPs with different particle sizes and morphologies using a solvothermal method. We found that the saturation magnetization intensity of these nanoparticles decreased as the particle size decreased. We then mixed these nanoparticles with CIPs to create bidisperse phase MRFs. We conducted a comparative analysis of these MRFs’ the yield stress, viscosity, storage modulus and sedimentation stability. Regarding particle size, CI-Fe_3_O_4_-MRF-250 nm demonstrated the best performance across all aspects. It exhibited a high yield stress of 12.7 kPa at 268.5 kA/m, a low zero-field viscosity of 1.23 Pa at 100 s^−1^ and good sedimentation stability of 53.8% after 1000 min. On the other hand, in terms of morphology, CI-Fe_3_O_4_-MRF-hexahedral exhibited the best performance. It showed a high yield stress of 13.6 kPa at 268.5 kA/m, a low zero-field viscosity of 1.27 Pa at 100 s^−1^ and excellent sedimentation stability of 56% after 1000 min. Furthermore, we investigated the mechanism behind the influence of particle size and morphology on MRF performance. We observed that appropriately sized nanoparticles formed a more stable magnetic chain structure. Polyhedral nanoparticles enhanced performance by providing a larger friction area. Additionally, smaller densities of nanoparticles improved sedimentation stability by reducing the overall density.This study offers valuable insights into novel MRFs’ design and practical application. It is particularly informative for modulating the properties of these smart materials by controlling the particle size, shape and composition.

## Figures and Tables

**Table 1:** The Fitting data of CI-MRF and CI-Fe_3_O_4_-MRF-10 nm.

Samples	Magnetic Field Strength	Herschel-Bulkley				Bingham			Casson		
	**kA/m**	$\tau_{y}$	**K**	η	** $R^{2}$ **	$\tau_{y}$	$\eta_{0}$	** $R^{2}$ **	$\tau_{y}$	$\eta_{\infty }$	** $R^{2}$ **
CI-MRF	13.4	33	155	0.16	0.99	187	1.47	0.64	165	0.37	0.83
	26.9	159	187	0.22	0.96	407	2.87	0.64	266	0.66	0.83
	67.2	1041	300	0.34	0.96	1330	16.19	0.76	1201	3.09	0.92
	134.3	3361	439	0.37	0.98	3918	16.67	0.81	3682	2.40	0.95
	268.5	9357	164	0.66	0.96	9553	28	0.91	9180	2.69	0.95
CI-Fe_3_O_4_-MRF-10 nm	13.4	52	161	0.15	0.99	206	1.41	0.63	185	0.32	0.82
	26.9	229	231	0.18	0.98	454	2.62	0.62	414	0.53	0.83
	67.2	1295	258	0.31	0.97	1556	7.10	0.71	1456	1.17	0.91
	134.3	4043	273	0.43	0.98	4376	14.67	0.83	4145	1.79	0.97
	268.5	10,246	367	0.49	0.98	10,730	27.25	0.88	10,294	2.56	0.99

## Data Availability

Data are contained within the article.

## Abbreviations

The following abbreviations are used in this manuscript:

MRFs	Magnetorheological Fluids
CI	Carbonyl Iron
NPs	Nanoparticles
CIPs	Carbonyl Iron Nanoparticles
SR	Sedimentation Ratio
